# Coolsecture: an easy-to-use and improved framework for cross-species Hi-C contact map comparison

**DOI:** 10.1093/bioinformatics/btag683

**Published:** 2026-09-17

**Authors:** Peng-Kai Zhu, Jiang-Qi Pan, Zhan-Chao Cheng, Jian Gao

**Affiliations:** Key Laboratory of National Forestry and Grassland Administration on Bamboo & Rattan, International Center for Bamboo and Rattan, Beijing 100102, China; Center for Agricultural Resources Research, Institute of Genetic and Developmental Biology, Chinese Academy of Sciences, Shijiazhuang 050022, China; Key Laboratory of National Forestry and Grassland Administration on Bamboo & Rattan, International Center for Bamboo and Rattan, Beijing 100102, China; Key Laboratory of National Forestry and Grassland Administration on Bamboo & Rattan, International Center for Bamboo and Rattan, Beijing 100102, China

## Abstract

**Summary:**

Cross-species Hi-C comparison remains challenging because existing workflows often rely on multiple scripts, heterogeneous I/O formats, and limited diagnostic support. We present coolsecture, a Python 3 command-line toolkit that integrates multiple contact-matrix and synteny formats with bidirectional lift-over and reciprocal consistency assessment, multi-resolution percentile-based comparison, diagnostic visualization, and cross-sample similarity analysis, providing a streamlined and reproducible framework for comparative Hi-C analysis.

**Availability and implementation:**

coolsecture is distributed under the GPL-3 license. Source code, Snakemake workflows, and documentation are freely available at https://github.com/pk-zhu/Coolsecture and are archived on Figshare at https://doi.org/10.6084/m9.figshare.30158440

## 1 Introduction

In eukaryotic genomes, 3D chromatin organization plays important roles in gene regulation, DNA replication, and genome stability (Sexton and Cavalli 2015, Xu *et al*. 2025). Hi-C has revealed chromatin organization across multiple scales, including chromosome territories, compartments, topologically associating domains (TADs), loops, and local interaction patterns (Oudelaar and Higgs 2021, Bienko 2023). Comparative Hi-C studies across species can therefore provide insights into the evolutionary conservation and divergence of genome folding, including changes associated with genome size, chromosome structure, and gene regulation (Krefting *et al*. 2018, Wang *et al*. 2018, 2021, Li *et al*. 2022). However, direct comparison of Hi-C contact maps across species remains technically challenging, particularly because homologous genomic regions must be mapped consistently and contact information must be represented in a comparable form across genomes (Nuriddinov and Fishman 2019, Eres and Gilad 2021).

Existing cross-species Hi-C comparisons have often focused on predefined structural features, such as TADs, chromatin loops, or domain boundaries, followed by homology-based comparison of their genomic positions (Li *et al*. 2022, Yang *et al*. 2025). Such feature-based approaches can be sensitive to the choice of calling algorithm, parameter settings, resolution, and synteny mapping. Complementary approaches therefore compare contact patterns more directly. Although C-InterSecture introduced statistical measures such as percentile-based background-adjusted distance (P-BAD) to move beyond simple boundary alignment (Nuriddinov and Fishman 2019), users still face the burden of managing multiple scripts, complex configuration files, and limited I/O and visualization standards (Nuriddinov and Fishman 2019).

To address these challenges and improve the reproducibility and user-friendliness of cross-species Hi-C analysis, we developed coolsecture, a framework that supports multiple input formats and directly processes contact matrices in .cool/.mcool and .hic formats. It integrates synteny-based bidirectional lift-over with consistency assessment, multi-resolution analysis, window-based comparison, distance binning, and statistical metrics such as P-BAD, while also providing standardized I/O interfaces and visualization support. The tool is designed to be simple and intuitive, enabling researchers to rapidly perform comparative 3D genome analyses across species or samples without being constrained by specific boundary-calling algorithms or preprocessing pipelines.

Compared with C-InterSecture, coolsecture provides several substantive extensions: (1) broader I/O compatibility, with support for both .cool/.mcool and .hic formats; (2) expanded synteny input support, accommodating both link-style files and UCSC .chain files; (3) bidirectional lift-over summaries and reciprocal consistency statistics; (4) multi-resolution analysis and stability summaries; (5) a HiCRep-inspired stratum-adjusted correlation coefficient (SCC)-like similarity score based on distance-stratified Pearson correlations (Yang *et al*. 2017); and (6) a modernized end-to-end workflow. Collectively, these features improve both reproducibility and interpretability.

## 2 Methods

### 2.1 Architecture and installation

Coolsecture is a modular Python package defined through a pyproject.toml file. A single CLI entry point, coolsecture, dispatches to subcommands, each of which is implemented in its own module. Optional dependencies include minimap2 (or MUMmer4) for genome alignment, samtools for FASTA indexing, hic2cool for processing .hic files, and Snakemake for workflow automation.

### 2.2 Standardized I/O and data initialization

Coolsecture accepts contact matrices in .cool, .mcool, and .hic formats. Cooler-format matrices are read directly, whereas .hic files are internally converted to Cooler format. This design supports Cooler-format matrices converted from the outputs of pipelines such as HiC-Pro (Servant *et al*. 2015), as well as .hic matrices generated by Juicer (Durand *et al*. 2016). For each processed resolution, the *prepare* subcommand produces two primary tables. The contact table records the chromosome, start and end coordinates, and global bin IDs of each pair of interacting bins, together with percentile ranks and their lower and upper bounds, per-bin coverage calculated by summing raw counts across retained pixels, and contact distance in bins. The distance-stratified statistics table reports the sample size, the 5th, 50th, and 95th percentiles, and 100 evenly spaced quantile reference values spanning the 0.5th to 99.5th percentiles. Cis contacts are stratified by genomic separation expressed in units of the matrix resolution, whereas trans contacts are assigned to distance bin −1 when included. Contact counts are normalized according to the convention of the selected bin-level normalization vector. For divisive normalization vectors, raw counts are divided by the product of the values for the two interacting bins; for the standard multiplicative Cooler weight vector, raw counts are multiplied by the product of the two bin weights. Pixels involving bins with non-finite or non-positive normalization values are excluded before distance-stratified percentile ranking.

Coolsecture then constructs an empirical distribution of normalized contact values within each distance stratum and assigns each retained pixel a percentile rank. Tied values are assigned midranks, while their lower and upper percentile-rank bounds are defined by the left and right edges of the corresponding tied block, respectively. These bounds retain the percentile-rank interval induced by tied contact values.

### 2.3 Lift-over and chain conversion

Coolsecture supports multiple synteny input formats, including link-style files and UCSC .chain files. Cross-species lift-over proceeds in three stages. (1) *asm2link* wraps minimap2 (Li 2021) or MUMmer4 (Marçais *et al*. 2018) to align the two assemblies and converts the resulting alignments into six-column synteny links that record the chromosome, start, and end coordinates in both the query and target genomes while preserving mapping orientation. (2) *link2mark* converts links generated by *asm2link*, other synteny tools, or .chain files into .mark files, with optional segment densification and gap-segment generation while preserving mapping orientation. (3) *liftcontacts* performs lift-over in both directions, merges the resulting mappings, and reports reciprocal consistency together with related summary statistics. For one-to-many mappings, target mappings of each source anchor are first grouped by target chromosome; mappings whose adjacent target bins are separated by no more than the specified aggregation frame are merged into the same local group. If a source contact subsequently yields multiple candidate target-group pairs, one candidate is retained according to the selected criterion: the smallest target percentile-rank uncertainty, the shortest target contact distance with cis contacts preferred over trans contacts, the highest product of the two target-bin coverages, or the highest summed remapping weight. When no additional ranking is requested, the first candidate encountered is retained. All other candidates are recorded as alternative mappings.

The *lift2matrix* subcommand converts the generated .liftContacts table into paired observed and target matrices in .cool or .hic format. The observed matrix stores source-species percentile scores, whereas the target matrix stores the corresponding mapped target-species percentile scores. Both matrices remain in the source-genome coordinate system and are represented on a 0–99 percentile scale, providing a unified representation for downstream visualization and quantitative comparison.

### 2.4 Metric computation and diagnostic visualization

The *metric* subcommand reports window-based statistics as bedGraph tracks and summary plots: (1) P-BAD scores; (2) log10 ratios of observed vs. target percentiles, with a small floor applied to each side to avoid division by zero; (3) stripe scores, the mean log10 ratio of observed vs. target percentiles within a one-sided window of size frame around each anchor; and (4) Pearson or Spearman correlations for matched pixels. The bedGraph outputs facilitate the identification of conserved or divergent regions and enable visualization in IGV or similar tools.

The *contact-stat* subcommand generates three diagnostic plots: (1) a percentile heatmap comparing percentile scores between species, (2) a heatmap of genomic distances for lifted-over locus pairs, and (3) a scatter plot of log2 genomic distance ratios vs. log2 percentile score ratios or contact frequency ratios, revealing contact reorganization beyond genomic distance changes. To maintain compatibility with the scatter plot visualization style used in C-InterSecture, we provide an option for its ratio scatter settings. These plots can be generated in PDF, PNG, or SVG format. In addition, the *plot-cross* subcommand creates heatmaps that visualize contacts within selected regions across species, enabling rapid visual comparison of regional contact patterns.

### 2.5 Cross-sample similarity scoring

The *similarity* subcommand calculates Pearson correlations within strata defined by genomic distance and combines them into a weighted average, with each stratum weighted by its number of contact pairs, to obtain the SCC-like score. Notably, unlike the *metric* subcommand, which evaluates local differences or correlations across specific regions, the *similarity* subcommand quantifies the overall concordance between the observed and target matrices at matched genomic coordinates. This enables quantitative comparison of overall contact-map similarity across species and samples.

### 2.6 Command-line interface and workflow integration

All functions are exposed through the CLI as coolsecture < subcommand>, eliminating the need to run separate scripts or edit INI files and providing concise descriptions of parameters. In addition to the core analysis subcommands, coolsecture provides a multiscale subcommand for summarizing P-BAD profiles across resolutions and assessing cross-resolution stability, together with a streamlined run-all subcommand with simplified default settings. The entire workflow can further be automated using the accompanying Snakemake file, which orchestrates the execution of link2mark, prepare, liftcontacts, metric, contact-stat, lift2matrix, and similarity across multiple species pairs (Fig. 1A), while writing outputs using predictable prefix-based naming conventions.

**Figure 1:**
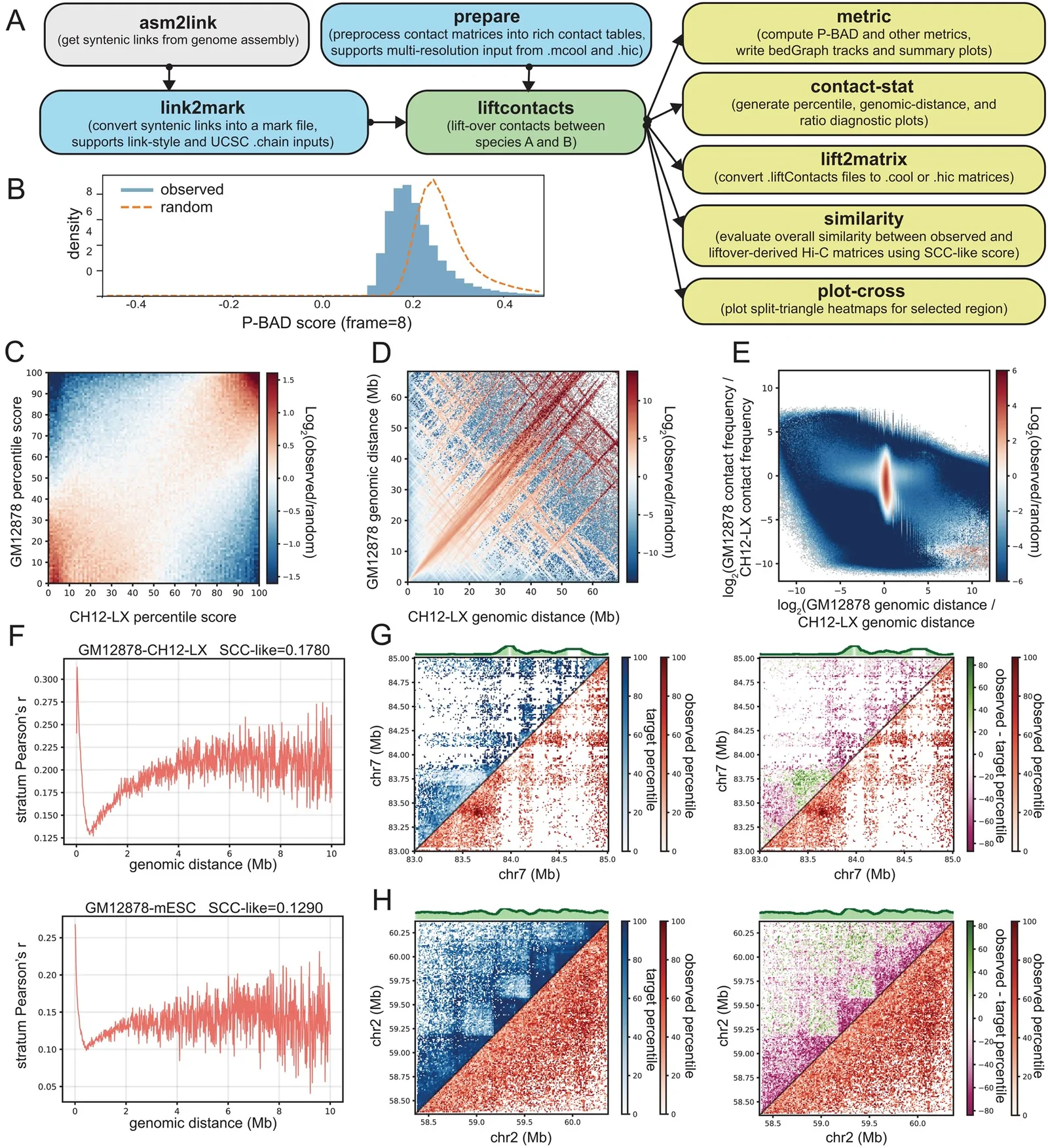
Cross-species Hi-C contact-map comparison and visualization using coolsecture. (A) Workflow of the coolsecture cross-species analysis. (B) Distribution of P-BAD scores (observed vs. randomized null). In the null, target-genome percentile ranks are shuffled across contacts while observed ranks are held fixed. (C–E) Comparative contact statistics for GM12878 and CH12-LX. (C) Contact strength percentile heatmap. (D) Contact genomic distance heatmap for lifted-over locus pairs. (E) Scatter plot of log2 genomic distance ratio against the log2 contact frequency ratio. Red denotes enrichment relative to the random background, whereas blue denotes depletion. (F) Overall similarity of the GM12878–CH12-LX and GM12878–mESC comparisons. (G, H) Representative plot-cross heatmaps of a conserved region (G) and a divergent region (H) in the GM12878-CH12-LX comparison. The left panel shows observed percentiles in the lower triangle and target percentiles in the upper triangle. The right panel shows observed percentiles in the lower triangle and percentile differences between observed and target in the upper triangle. A genomic track aligned above the heatmaps indicates the P-BAD score for the corresponding genomic region.

## 3 Results

To demonstrate the functionality of coolsecture, we compared publicly available .hic matrices from the 4DN data portal (https://data.4dnucleome.org/), including the human GM12878 cell line (4DNFI1UEG1HD) and the mouse CH12-LX cell line (4DNFI8KBXYNL). We further evaluated overall similarity across different cell types using GM12878 and the mouse mESC cell line (4DNFIU8AF5ZY). Human-to-mouse synteny mapping was performed using UCSC .chain files (https://hgdownload.soe.ucsc.edu/goldenPath/hg38/liftOver/). Each matrix was processed at 10 kb resolution using the *prepare* subcommand. The synteny files were converted to .mark format using *link2mark*, followed by bidirectional lift-over and consistency assessment with *liftcontacts*. Figure 1 summarizes the workflow outputs, including the global P-BAD distribution (Fig. 1B), diagnostic comparisons (Fig. 1C–E), overall similarity assessed using the SCC-like score (Fig. 1F), and locus-level visualizations (Fig. 1G–H).

The *metric* subcommand shows the distribution of windowed P-BAD scores for the human–mouse comparison (Fig. 1B). The observed distribution peaks at approximately 0.2, whereas the randomized null distribution is shifted toward higher values. In addition, the observed data exhibit a heavier high-score tail, indicating an excess of loci with stronger deviation than expected under the randomized null.

The diagnostic outputs generated by the *contact-stat* subcommand further support the interpretability of the percentile-based scoring scheme. The contact strength percentile heatmap (Fig. 1C) shows enrichment in the lowest and highest percentile ranges relative to the random background, indicating non-random correspondence between contact-strength percentiles across the two species.

The contact genomic distance heatmap (Fig. 1D) displays the joint distribution of distances for lifted-over locus pairs between source and target species. Enrichment near the diagonal indicates that a subset of syntenic loci exhibits similar genomic distances between species, whereas the slight offset from the diagonal reflects larger genomic distances in human than in mouse. The ratio scatter plot (Fig. 1E), generated using the C-InterSecture standard setting, shows that most matched contacts cluster near a log2 genomic-distance ratio of zero, whereas variation in contact frequency is more pronounced, indicating that many loci retain similar genomic distances but differ in contact frequency between species.

Using the *similarity* subcommand, we calculated SCC-like scores to assess the correlation between the lifted-over human GM12878 matrix and two mouse cell types. We observed that GM12878–CH12-LX showed a higher SCC-like score (0.1780) than GM12878–mESC (0.1290). Thus, the GM12878 matrix showed greater concordance with CH12-LX than with mESC. This pattern is consistent with the shared B-cell/lymphoid lineage context of GM12878 and CH12-LX (Choudhary *et al*. 2020), illustrating that the SCC-like score can capture expected differences in cross-sample contact-map similarity.

At the locus level, *plot-cross* heatmaps further demonstrate the ability of coolsecture to distinguish between conserved and divergent regions. For the conserved region (Fig. 1G), the observed and target matrices display broadly similar percentile patterns, and the difference map shows only relatively weak deviation signals, indicating that the local contact structure is relatively well conserved between the two species. In contrast, for the divergent region (Fig. 1H), the observed and target patterns differ more markedly, and the difference map exhibits continuous deviation signals of larger magnitude, suggesting more pronounced cross-species structural divergence in this region. These results indicate that the framework can effectively visualize the conservation and divergence of local contact patterns at the locus level.

## 4 Discussion

Coolsecture provides a more standardized and extensible framework for cross-species Hi-C comparison. The tool does not depend on a specific compartment, TAD, or loop caller, and can therefore be flexibly integrated with users’ existing downstream annotation workflows. In addition, domain annotations from the source species can be projected onto the target species using *roughlift*, thereby supporting cross-species structural comparisons. In practice, higher-resolution matrices may reveal more localized contact differences but can also reduce cross-matrix similarity because of increased sparsity, noise, and fewer comparable contacts. Additionally, computational cost is determined mainly by the size and sparsity of the input contact matrices, as well as by the total number of remapped contacts after lift-over; accordingly, larger genomes will generally require greater runtime and memory resources. Future work will extend support for multi-way comparisons and strengthen integration with other omics data and additional validation tasks.

## 5 Conclusion

We developed coolsecture, a reproducible and user-friendly framework for cross-species Hi-C comparison. By combining standardized I/O support, bidirectional lift-over, similarity assessment, and integrated diagnostic visualization within a single workflow, coolsecture extends earlier comparative Hi-C pipelines in both functionality and usability. Using a human–mouse example, we show that the framework captures both global and locus-specific variation in chromatin contact patterns and enables quantitative comparison of contact-map similarity across samples. Together, these features make coolsecture a robust and extensible tool for comparative 3D genomics.

## Data Availability

No new biological datasets were generated in this study. The Hi-C datasets used for demonstrating coolsecture are publicly available from the 4D Nucleome Data Portal (https://data.4dnucleome.org/) under accession numbers 4DNFI1UEG1HD (human GM12878), 4DNFI8KBXYNL (mouse CH12-LX), and 4DNFIU8AF5ZY (mouse mESC). Human-to-mouse chain files are publicly available from the UCSC Genome Browser (https://hgdownload.soe.ucsc.edu/goldenPath/hg38/liftOver/). The source code, workflows, and documentation for coolsecture are available at https://github.com/pk-zhu/Coolsecture and are permanently archived on Figshare at https://doi.org/10.6084/m9.figshare.30158440.
